# Clinical Impact of Preexisting Right Bundle Branch Block after Transcatheter Aortic Valve Replacement: A Systematic Review and Meta-Analysis

**DOI:** 10.1155/2020/1789516

**Published:** 2020-07-21

**Authors:** Garly R. Saint Croix, Spencer C. Lacy, Hakop Hrachian, Nirat Beohar

**Affiliations:** ^1^Columbia University Division of Cardiology, Mount Sinai Medical Center, Miami Beach, FL, USA; ^2^Miller School of Medicine, The University of Miami, Miami, FL, USA

## Abstract

**Introduction:**

Transcatheter aortic valve replacement (TAVR) is now the treatment of choice for patients with severe aortic stenosis regardless of their surgical risk. Right bundle branch block (RBBB) can be a predictor for development of significant atrioventricular (AV) block after TAVR, requiring permanent pacemaker implantation (PPI). However, data related to the risk of PPI requirement with preexisting RBBB is scarce. Hence, this systematic review and meta-analysis aims to assess clinical outcomes of patients undergoing TAVR with RBBB on preexisting electrocardiogram.

**Methods:**

We performed a systematic literature review to identify randomized and nonrandomized clinical studies that reported any clinical impact of patients undergoing TAVR with preexisting RBBB. A total of eight databases including PubMed (Medline), Embase, Cochrane Library, ACP Journal Club, Scopus, DARE, and Ovid containing articles from January 2000 to May 2020 were analyzed.

**Results:**

We identified and screened 224 potential eligible publications through the databases and found 14 relevant clinical trials for a total of 15,319 participants. There was an increased 30-day pacemaker implantation rate of 38.1% in the RBBB group compared to 11.4% in the no RBBB group with a risk ratio of 3.56 (RR 3.56 (95% CI 3.21–3.93, *p* < 0.01)). There was an increased 30-day all-cause mortality in the RBBB group of 9.5% compared with 6.3% in the no RBBB group with an odds ratio of 1.60 (OR 1.60 (95% CI 1.14–2.25, *p* < 0.01)).

**Conclusion:**

This study indicates that patients with preexisting RBBB have higher incidence of PPI and all-cause mortality after TAVR compared with patients without RBBB. Further trials are needed to compare the clinical outcomes based on TAVR valve types and assess the benefit of PPI in patients with new-onset RBBB after TAVR.

## 1. Introduction

Transcatheter aortic valve replacement (TAVR) has revolutionized the current era of modern medicine by becoming the treatment of choice for patients with symptomatic severe aortic stenosis regardless of their surgical risk [1]. However, the high frequency of conduction disturbances, such as left bundle branch block (LBBB) and atrioventricular block, and the subsequent need for permanent pacemaker implantation (PPI) remain a challenge [2, 3]. Preexisting right bundle branch block (RBBB) has been established as a risk factor for PPI after TAVR [4, 5]. Preexisting RBBB in the general population and in patients with heart disease has been associated with increased risk of mortality [6]. However, data on the prognostic impact of preexisting RBBB on clinical outcomes after TAVR is limited. This systematic review and meta-analysis evaluates the impact of preexisting RBBB on clinical outcomes in patients undergoing TAVR.

## 2. Methods

The main objective of this review was to assess if preexisting RBBB increased the risk of having PPI after TAVR. We used the Preferred Reporting Items for Systematic Reviews and Meta-Analyses (PRISMA) statement extension for network meta-analysis. The PRISMA flow diagram was used to depict the four phases of the review including identification, screening, eligibility, and inclusion. The PRISMA statement contains a checklist of 27 items required of systematic reviews and meta-analyses. The review was not registered a priori. No ethical approval was required since this meta-analysis uses only public published data.

### 2.1. Search Strategy

We performed a systematic literature review to identify randomized and nonrandomized clinical studies that reported any clinical impact of patients undergoing TAVR with preexisting RBBB. Searches were limited to peer-reviewed primary research articles published in English, French, and Spanish up to May 17^th^, 2020. This research involved human subjects and described the clinical impact of RBBB on patients who underwent TAVR. We developed the search strategy according to available guidance from the Cochrane Collaboration.

The search strategy in MEDLINE explored Medical Subject Heading (MeSH) terms related to patients with TAVR and history of preexisting RBBB. The following search strategy was applied to search MEDLINE and we adapted it for the other databases: (“transcatheter aortic valve replacement”[MeSH Terms] OR (“transcatheter”[All Fields] AND “aortic”[All Fields] AND “valve”[All Fields] AND “replacement”[All Fields] AND “implantation”[All Fields])) OR “transcatheter aortic valve replacement”[All Fields]) AND (“bundle-branch block”[MeSH Terms] OR (“bundle-branch”[All Fields] AND “block”[All Fields]) OR “bundle-branch block”[All Fields] OR (“right”[All Fields] AND “bundle”[All Fields] AND “branch”[All Fields] AND “block”[All Fields]) OR “right bundle branch block”[All Fields]). The articles found to be relevant during the hand search were stored in EndNote. Selected articles underwent full evaluation to assess their potential inclusion in the systematic review.

### 2.2. Study Selection

Articles were selected for inclusion based on predefined criteria, which included age, sex, TAVR, and preexisting RBBB, and the primary or secondary outcomes being mortality and clinical outcomes. Exclusion criteria were patients with LBBB and patients with normal sinus rhythm. We excluded case reports and studies with fewer than 10 subjects.

Two authors (GS, SL) independently read the trials and screened the abstracts to choose potentially relevant articles. Risk of bias in the studies was assessed at an individual level of each study. Selected articles underwent full evaluation to assess their potential inclusion in the systematic review.

### 2.3. Statistical Analysis

Data were analyzed using Review Manager Software 5.3. We used fixed effects to assess the combined risk estimates according to I2 statistics. Analysis to determine sensitivity and publication bias was detected by funnel plots. *p* < 0.05 was considered statistically significant.

## 3. Results

### 3.1. Literature Search

**Figure 1:**
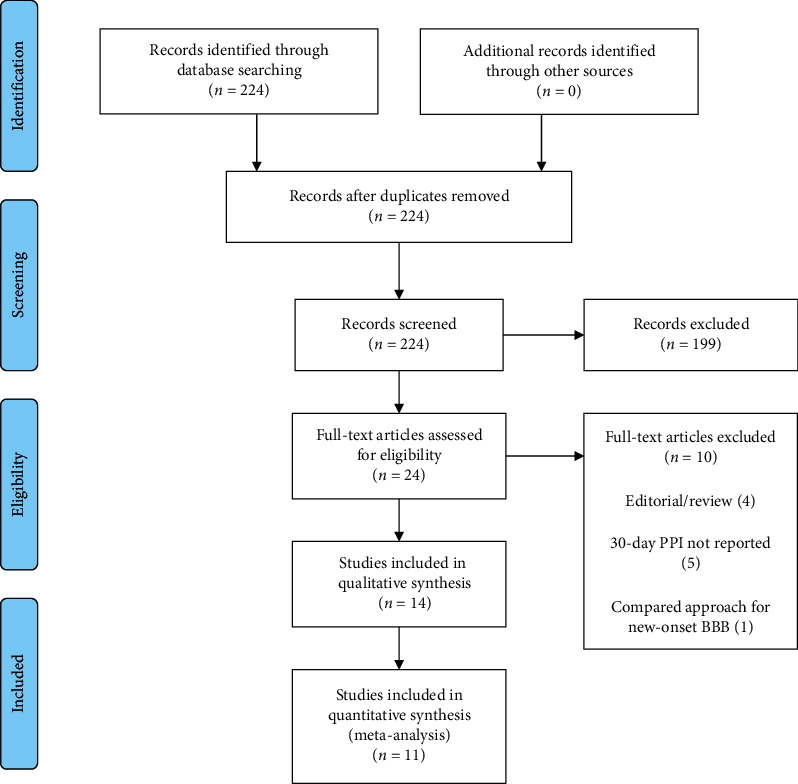
Flowchart of the included studies.

Our search yielded 224 abstracts. We excluded 199 studies at the abstract level and selected 24 full-text articles for detailed assessment; 14 studies were ultimately included in our systematic review and 11 studies were included in our meta-analysis.  Figure 1 describes the flowchart of included studies.

### 3.2. Baseline Characteristics of the Studies

Table 1 shows the baseline characteristics of the included studies. All studies were published between 2010 and 2020. The 14 studies included 15,319 patients with 1,654 cases of preexisting RBBB. In nine of the included studies, preexisting RBBB was retrospectively identified as a risk factor for PPI. Therefore, baseline characteristics for patients with preexisting RBBB were not reported in these nine studies. For studies that did report characteristics for both RBBB and non-RBBB patients, the median age of the participants was 82.0 IQR (81.4–84.0). The median percentage of men was 49.1 IQR (39.2–58.4). For studies that reported these selected risk factors, the median percentage of hypertension was 76.1 IQR (74.8–81.9), the median percentage of diabetes was 30.2 IQR (28.9–32.8), the median BMI average was 26.7 IQR (24.3–27.1), the median percentage of coronary heart disease was 56.4 IQR (34.4–64.5), the median percentage of heart failure greater than or equal to New York Heart Association Class III was 74.7 IQR (47–77), and the median percentage of chronic obstructive pulmonary disease was 21.6 IQR (19.2–27.7). Current smoking percentage was 20.6% in the preexisting RBBB group and 19.9% in the no RBBB group for the one study that reported this risk factor. Racial characteristics were not reported by the included studies that used separate preexisting RBBB groups. Multiple centers were used by eight of the included studies and Europe, North America, South America, Japan, and Israel were the geographic regions represented.

### 3.3. PPI in Patients with Preexisting RBBB after TAVR

Auffrett et al., Husser et al., van Gils et al., Tovia-Brodie et al., and Watanabe et al. reported various clinical outcomes in patients with preexisting RBBB after TAVR as summarized in Table 2 [7–11]. Auffrett et al. found patients with preexisting RBBB to have higher 30-day PPI rates (40.1% vs. 13.5%; *p* < 0.001) [7]. Husser et al. reported a 30-day PPI rate of 39.2% in patients with preexisting RBBB after TAVR and found the ACURATE neo (Boston Scientific, Marlborough, Massachusetts) to have a lower rate of PPI when compared with the Edwards Sapien 3 (Edwards Lifesciences, Irvine, California) [8]. Van Gils et al. reported a 30-day PPI rate of 41.0% in patients with preexisting RBBB after TAVR and found the Boston Scientific Lotus (Boston Scientific, Marlborough, Massachusetts) to have the highest rate of PPI and the Edwards Sapien 3 and XT (Edwards Lifesciences, Irvine, California) to have the lowest rate of PPI [9]. Watanabe et al. found patients with preexisting RBBB to have higher 30-day rates of PPI (17.6% vs. 2.9%; *p* < 0.01) [10]. Tovia-Brodie et al. did not report a 30-day PPI rate as they compared whether prophylactic PPI improved outcomes in patients with preexisting RBBB [11].

The nine remaining studies identified risk factors for 30-day PPI as summarized in Table 2 [12–20]. Meduri et al. identified preexisting RBBB, female sex, and depth of implantation to be a risk factors for 30-day PPI in their retrospective review of the REPRISE III (The Repositionable Percutaneous Replacement of Stenotic Aortic Valve through Implantation of Lotus Valve System-Randomized Clinical Evaluation) trial [12]. Nazif et al. identified preexisting RBBB, prosthesis to left ventricular outflow tract diameter ratio, and left ventricular end-diastolic diameter as risk factors for 30-day PPI in their retrospective review of the PARTNER (Placement of AoRtic TraNscathetER Valves) trial [15]. Dhakal et al., Roten et al., Erkapic et al., Koos et al., and Fraccarro et al. all identified preexisting RBBB as a risk factor for PPI after TAVR in their retrospective single-center studies [13, 16–19]. Bagur et al. found preexisting RBBB as the only risk factor for PPI after TAVR in their comparison to surgical aortic valve replacement [14]. Guetta el al. identified preexisting RBBB and deep valve implantation as risk factors for PPI after TAVR in their retrospective review at three referral centers in Israel [20].

**Figure 2:**
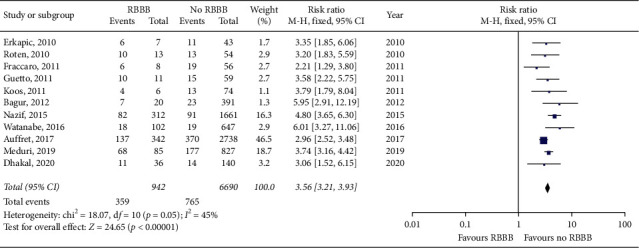
Forest plot of 30-day PPI rates in patients with and without preexisting RBBB after TAVR.

**Figure 3:**
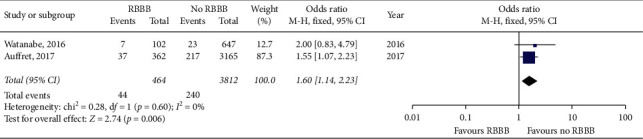
Forest plot of 30-day all-cause mortality rate in patients with and without preexisting RBBB after TAVR.

Meta-analysis of the included studies revealed a higher 30-day PPI rate of 38.1% in patients with preexisting RBBB when compared to a 30-day PPI rate of 11.4% in patients with no RBBB. This is a statistically significant increase in 30-day PPI rate in patients with preexisting RBBB with a risk ratio of 3.56 (95% CI 3.21–3.93, *p* < 0.01). The forest plot for 30-day PPI rate is shown in Figure 2. Husser et al., Tovia-Brodie et al., and van Gils et al. results were not included in the 30-day PPI meta-analysis as these studies only included patients with preexisting RBBB and made no comparisons to patients without RBBB. In the two included studies that reported 30-day mortality as an outcome, meta-analysis revealed a higher 30-day mortality rate of 9.5% in patients with preexisting RBBB compared to a 30-day mortality rate of 6.3% in patients with no RBBB. This is a statistically significant increase in 30-day mortality in patients with preexisting RBBB with a risk ratio of 1.60 (95% CI 1.14–2.25, *p* < 0.01). The forest plot for 30-day morality rate is shown in Figure 3.

## 4. Discussion

This is the first systematic review and meta-analysis to demonstrate the impact of preexisting RBBB on new pacemaker implantation after TAVR. Our findings are derived from 14 studies reporting clinical outcomes in a total of 1,654 patients with preexisting RBBB after TAVR. The incidence of new PPI was significantly increased in patients with preexisting RBBB after TAVR. Increased all-cause and cardiovascular mortality has been demonstrated in patients with preexisting RBBB after TAVR.

The prognostic value of RBBB has shown mixed results in previous studies with healthy participants and with patients with heart disease [21–28]. Bussink et al. found RBBB to be associated with increased all-cause and cardiovascular mortality in both men and women from the general population [21]. Abdel-Qadir et al. found no prognostic value of RBBB in patients hospitalized with heart failure; however, Barshehet et al. found RBBB to be associated with increased long-term mortality risk in hospitalized patients with systolic heart failure [22, 23]. Melgaregjo-Moreno et al. found new permanent RBBB to be associated with increased 30-day and seven-year mortality in patients with acute myocardial infarction [24]. Wong et al. found RBBB accompanying anterior acute myocardial infarction to be associated with increased 30-day mortality [28]. Long-term epidemiological studies in men from the general population have found a higher incidence of RBBB with aging, hypertension, and diabetes mellitus [25, 26]. Zhang et al. found RBBB in women with cardiovascular disease to be associated with an increased risk of coronary heart disease death over 14 years of follow-up. However, RBBB in women free of cardiovascular disease was not associated with increased mortality [27]. Meta-analysis completed by Xiong et al. found RBBB to be associated with an increased risk of mortality in the general population and in patients with heart disease [6]. The exact mechanism by which RBBB increases mortality has not been elucidated, although underlying conduction system disease can predispose patients to various arrhythmias. The association of RBBB with decreased left ventricular ejection fraction in patients with prior myocardial infarction or heart failure may provide a clue towards the underlying mechanism [23, 28]. The various comorbidities and underlying heart disease in patients with RBBB may also explain the increased mortality.

A previous meta-analysis by Siontis et al. demonstrated the significance of RBBB in the requirement for PPI after TAVR. Male sex, first-degree AV block, left anterior hemiblock, and intraprocedural AV block were also found to be predictive of PPI after TAVR [4]. The newer studies highlighted in this meta-analysis emerged to specifically focus on the clinical impact of preexisting RBBB after TAVR, which is important for improving patient outcomes [7–10].

In TAVR, conduction disturbances are a common complication likely due to direct insult to the left bundle branch because of the anatomical relationship between the aortic annulus and the conduction system [29]. New-onset LBBB develops in 5% to 65% of patients undergoing TAVR, and its persistence can result in PPI in 15% to 20% of cases [29]. New-onset LBBB after TAVR has been associated with increased risk of cardiac death and PPI at one year follow-up [30]. Preexisting RBBB with new-onset LBBB after TAVR will usually generate PPI during the index hospitalization. Chorianopoulos et al. demonstrated that postprocedural bradyarrhythmias develop in 36.2% of patients after TAVR with 3.8% remaining >96 hours after TAVR. Preexisting RBBB was found to be the only predictor of postprocedural bradyarrhythmias [5]. Late-onset new LBBB >3 months after TAVR is a rare complication seen in only 0.8% of patients [31]. Development of high degree AV block is a common complication seen in up to 58.8% of patients with preexisting BBB or BBB occurring during TAVR [32]. These late conduction disorders in patients with preexisting RBBB can lead to cardiac complications such as heart failure and sudden cardiac death.

This systematic review and meta-analysis reveals increased PPI, all-cause mortality, and cardiac mortality in patients with preexisting RBBB after TAVR. This is clinically significant given the recent trend towards early discharge after TAVR [33]. Patients with preexisting RBBB may need additional monitoring after TAVR to detect conduction disturbances and ensure safe discharge. Early electrocardiographic monitoring may be beneficial as part of the TAVR work-up as Urena et al. found that newly diagnosed preprocedural arrhythmias are common and associated with higher rates of PPI after the procedure [34]. Additional strategies for managing preexisting RBBB in patients undergoing TAVR may emerge as we understand more about conduction disturbances following TAVR.

Preexisting RBBB is a common underlying conduction disturbance in patients undergoing TAVR and is associated with increased risk of PPI at 30 days and all-cause and cardiovascular mortality. Future studies will be needed to evaluate optimal management of patients with preexisting RBBB undergoing TAVR. Larger prospective studies are needed to investigate the optimal timing for PPI after TAVR and to evaluate prophylactic PPI in patients with preexisting RBBB prior to TAVR. Larger prospective studies are needed to investigate strategies for early detection of conduction disturbances in patients with preexisting RBBB. Until more data is available, there are many multicenter and literature-based decisional algorithms to guide PPI decision-making [35]. Careful monitoring to detect arrhythmias after TAVR may be necessary to improve clinical outcomes in patients with preexisting RBBB.

## 5. Limitations

The limitations for this systematic review and meta-analysis are influenced by the limitations of the included studies. Auffrett et al. used a nonrandomized study design that may lead to confounders influencing the relationship between preexisting RBBB and outcomes [7]. The studies by Husser et al. and van Gils et al. are limited by their observational design [8, 9]. Tovia-Brodie et al. used a single-center retrospective design and did not use randomization to determine prophylactic pacemaker implantation [11]. The results from Watanabe et al. are limited by the relatively small size of the cohort (*n* = 749) and the relatively short median follow-up of 16 months [10]. The retrospective studies of existing data are subject to all of the limitations inherent to this study design [12–20]. Availability of specific data points, such as medication that could influence cardiac conduction, is a common limitation for retrospective studies. Roten et al., Erkapic et al., Koos et al., Fraccaro et al., and Guetta et al. all had small sample sizes of less than 100 patients in their studies [16–20]. All of the included studies are likely influenced by between-center variability and the lack of centralized independent assessment of procedural results and outcomes. The various valve types used in each study likely influence the generalizability of the aggregate data as specific valve types have shown different rates of procedural complications.

## 6. Conclusion

Conduction issues after TAVR continue to remain a common complication during the management of severe aortic stenosis. This current systematic review and meta-analysis indicates that patients with preexisting RBBB have a higher incidence of PPI and all-cause mortality after 30 days after TAVR compared with patients without RBBB. Further trials are needed to compare the clinical outcomes based on TAVR valve types and assess the benefit of PPI in patients with RBBB after TAVR. In addition, understanding the progression and prevention of electrical conduction disease are necessary for appropriate risk stratification, interventional strategy, and avoidance of pacemaker implantation.

## Figures and Tables

**Table 1:** Baseline characteristics of the included studies.

Study author, year	Region	Sample size	RBBB status	Number of patients	Mean age (years)	Men (%)	HTN (%)	Smoking (%)	DM (%)	BMI (kg/m^2^)	CHD (%)	NYHA > III (%)	COPD (%)
Watanabe et al., 2016	Japan	749	RBBB	102	85.0 (81.0–89.0)	39.2	80.4	20.6	26.4	22.2 (19.5–24.9)	28.0	52	21.6
			No RBBB	647	85.0 (82.0–88.0)	32.9	74.8	19.9	25.0	21.7 (19.3–24.1)	24.2	47	20.2
van Gils et al., 2017	Europe	2,845	RBBB	306	83 ± 7	63	NR	NR	30	27 ± 5	NR	77	30

Auffrett et al., 2017	Europe, Canada, S. America	3,527	RBBB	362	81.7 ± 7.3	58.4	74.7	NR	28.9	27.2 ± 5.2	59.2	74.7	29.9
			No RBBB	3165	81.4 ± 7.6	49.1	77.2	NR	30.2	26.7 ± 5.1	53.5	74.7	25.5

Husser et al., 2019	Germany and Switzerland	4,305	RBBB -S3	198	82.0 (78.0–85.0)	27.8	86.4	NR	32.8	26.4 (23.7–29.8)	68.2	78.3	18.2
			RBBB -NEO	98	81.0 (78.0–84.0)	39.8	92.9	NR	32.7	27.9 (24.7–31.0)	66.3	79.6	14.3

Tovia-Brodie et al., 2020	Israel	90	RBBB	50	81 ± 8	58	70	NR	34	NR	NR	18	NR
			RBBB -PM	40	84 ± 6	75	75	NR	43	NR	NR	20	NR

Meduri et al., 2019	N. America, Europe, Australia	912	RBBB	85	NR	NR	NR	NR	NR	NR	NR	NR	NR
			No RBBB	827	NR	NR	NR	NR	NR	NR	NR	NR	NR

Dhakal et al. 2020	Arizona, USA	176	RBBB	36	NR	NR	NR	NR	NR	NR	NR	NR	NR
			No RBBB	140	NR	NR	NR	NR	NR	NR	NR	NR	NR

Bagur et al. 2012	Canada	411	RBBB	20	NR	NR	NR	NR	NR	NR	NR	NR	NR
			No RBBB	391	NR	NR	NR	NR	NR	NR	NR	NR	NR

Nazif et al. 2015	United States, Canada, Germany	1,973	RBBB	312	NR	NR	NR	NR	NR	NR	NR	NR	NR
			No RBBB	1661	NR	NR	NR	NR	NR	NR	NR	NR	NR

Roten et al. 2010	Switzerland	67	RBBB	13	NR	NR	NR	NR	NR	NR	NR	NR	NR
			No RBBB	54	NR	NR	NR	NR	NR	NR	NR	NR	NR

Erkapic et al. 2010	Germany	50	RBBB	7	NR	NR	NR	NR	NR	NR	NR	NR	NR
			No RBBB	43	NR	NR	NR	NR	NR	NR	NR	NR	NR

Koos et al. 2011	Germany	80	RBBB	6	NR	NR	NR	NR	NR	NR	NR	NR	NR
			No RBBB	74	NR	NR	NR	NR	NR	NR	NR	NR	NR

Fraccaro et al. 2011	Italy	64	RBBB	8	NR	NR	NR	NR	NR	NR	NR	NR	NR
			No RBBB	56	NR	NR	NR	NR	NR	NR	NR	NR	NR

Guetta et al. 2011	Israel	70	RBBB	11	NR	NR	NR	NR	NR	NR	NR	NR	NR
			No RBBB	59	NR	NR	NR	NR	NR	NR	NR	NR	NR

Values are mean ± SD, median (interquartile range), or *n* (%). NR: not reported; HTN: hypertension; DM: diabetes mellitus; CHD: coronary heart disease; RBBB: right bundle branch block; BMI: body mass index; NYHA: New York Heart Association; COPD: chronic obstructive lung disease.

**Table 2:** Outcomes of patients with preexisting RBBB after TAVR summary table.

References	Year	Region	Centers	Patients w/RBBB	Valves	Primary outcome	Other outcomes
Watanabe et al.	2016	Japan	8	102	ES-XT	Various clinical outcomes	PPI, mortality, bleeding, etc.


van Gils et al.	2017	Europe	4	306	CoreValve ES-XT ES-3 Lotus	PPI within 30 days	New onset conduction disturbances

Auffrett et al.	2017	Europe, Canada, S. America	Not reported	362	Not reported	All-cause mortality	CV death, SCD, PPI

Husser et al.	2019	Germany and Switzerland	7	296	Neo ES-3	PPI within 30 days	Device failure

Tovia-Brodie et al.	2020	Israel	1	90	CoreValve ES-3 ES-XT Evolute R Lotus	Outcomes comparison for prophylactic PM	Predictors for pacing

Meduri et al.	2019	N. America, Europe, Australia	55	85	CoreValve Lotus	PPI within 30 days	Predictors for pacing, mortality, stroke, rehospitalization

Dhakal et al.	2020	Arizona, USA	1	36	Not reported, balloon expandable and self-expanding	PPI within 30 days	Predictors for pacing

Bagur et al.	2012	Canada	3	20	CE ES ES-XT	PPI within 30 days	Predictors for pacing

Naziif et al.	2015	United States, Canada, Germany	21	312	ES	PPI within 30 days	Predictors for pacing

Roten et al.	2010	Switzerland	1	13	CoreValve	PPI within 30 days	Predictors for pacing
					ES		

Erkapic et al.	2010	Germany	1	7	CoreValve	PPI within 30 days	Predictors for pacing
					ES		

Koos et al.	2011	Germany	1	6	CoreValve	PPI within 30 days	Predictors for pacing
					ES		

Fraccaro et al.	2011	Italy	1	8	CoreValve	PPI within 30 days	Predictors for pacing

Guetta et al.	2011	Israel	3	11	CoreValve	PPI within 30 days	Predictors for pacing

PPI: permanent pacemaker implantation; SCD: sudden cardiac death; CV: cardiovascular; ES-XT: Edwards SAPIEN XT; ES-3: Edwards SAPIEN 3; ES: Edwards SAPIEN; CE: Cribier-Edwards.

## Data Availability

The data used to support the findings of this study are included within the article.
